# Supplementary figures and images for: A human coronavirus evolves antigenically to escape antibody immunity
Source: PLoS Pathog. 2021 Apr 8;17(4):e1009453. doi: 10.1371/journal.ppat.1009453 (PMC8031418; doi:10.1371/journal.ppat.1009453)

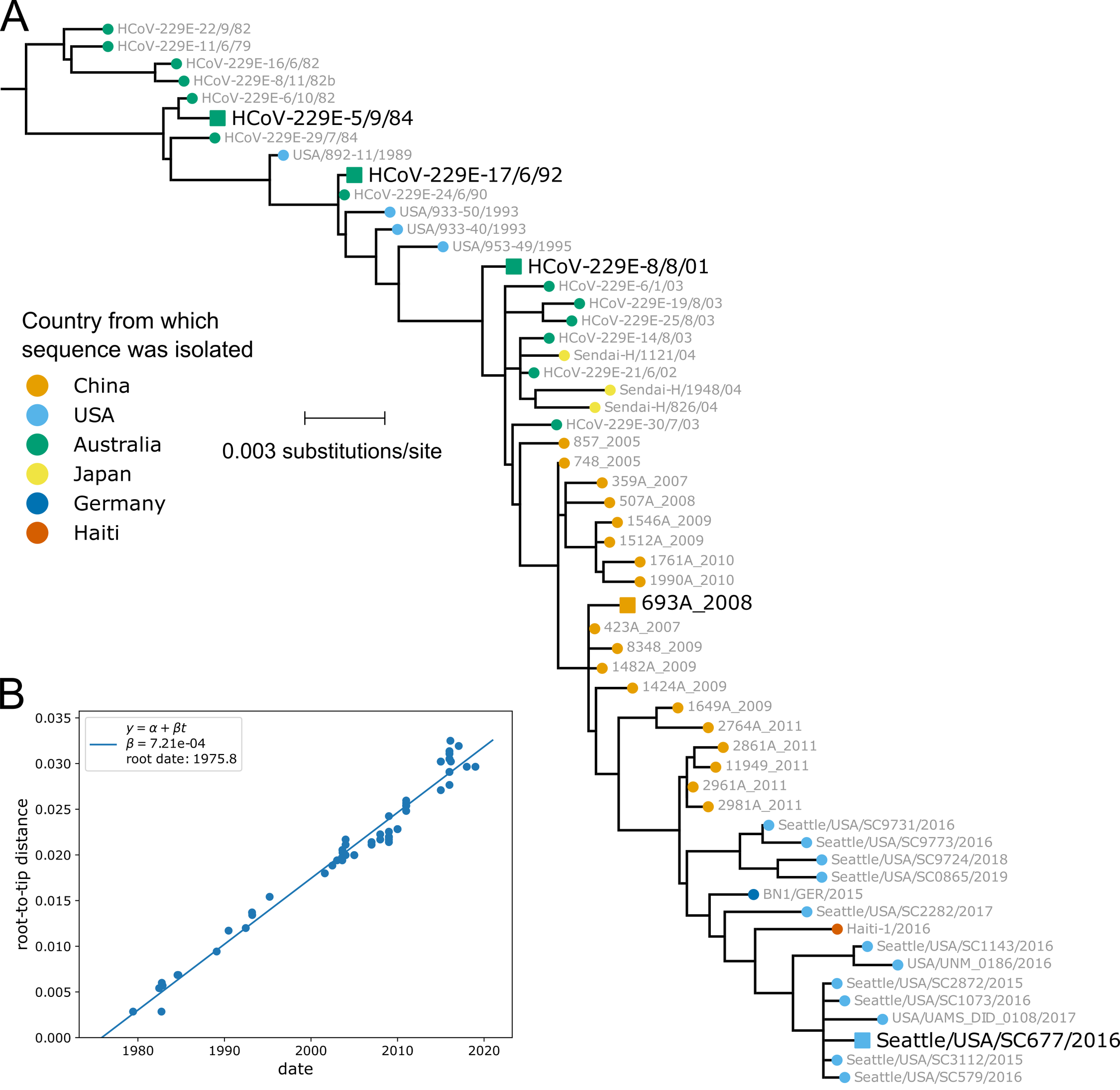

Supplement: S1 Fig — (A) Phylogenetic tree exactly like that in Fig 1 but with branch lengths proportional to divergence (not re-scaled based on tip isolation date). (B) A plot produced by TreeTime showing the correlation between the distance of tip nodes from the root and sampling date. The fact that all points fall on a line indicates that the evolution is clock-like. (TIF) [file ppat.1009453.s001.tif]

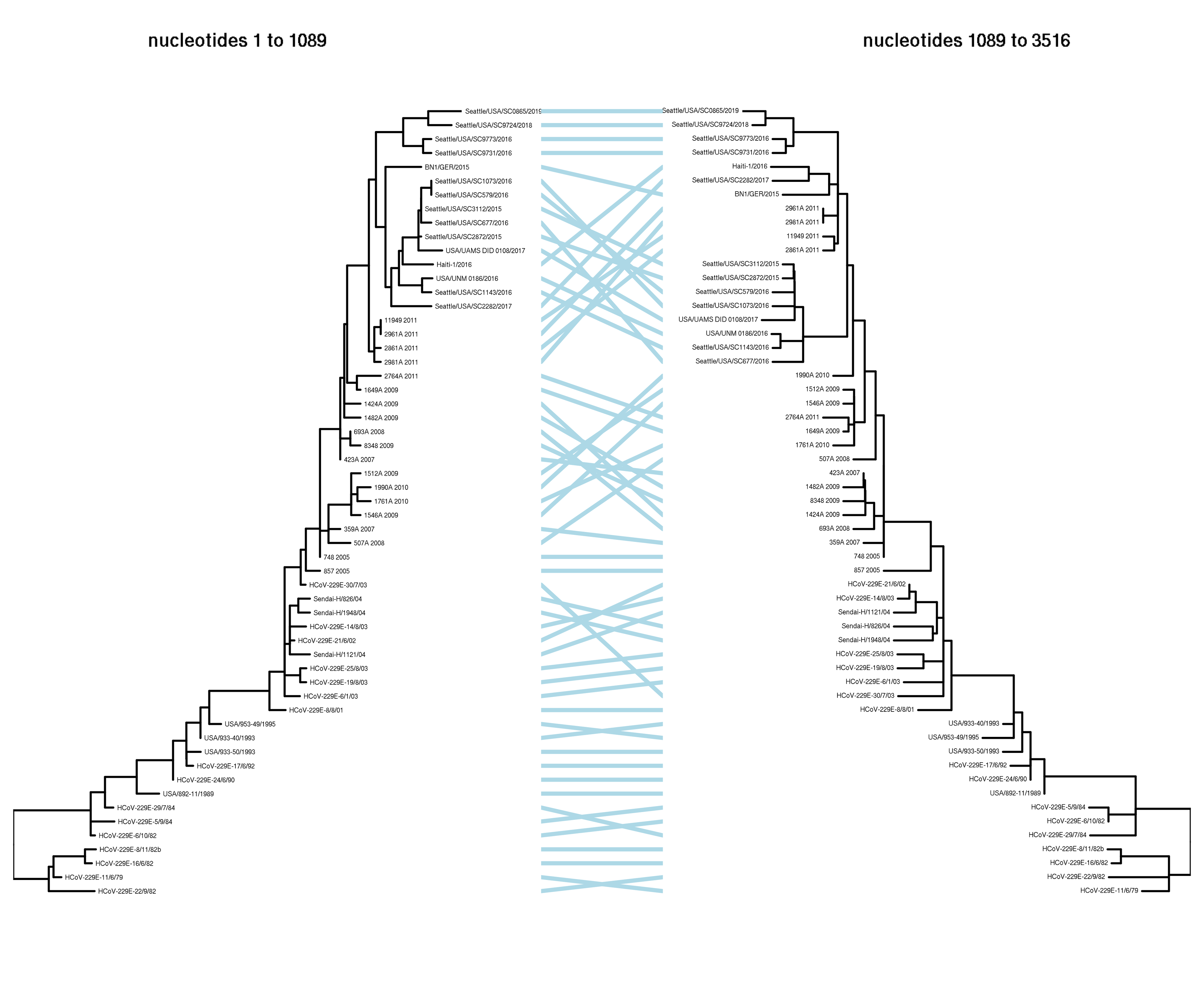

Supplement: S2 Fig — Specifically, GARD was used to analyze the same set of 229E spike sequences used in Fig 1 with a nucleotide substitution model and three gamma-distributed rate classes. The best-fitting model had a single recombination breakpoint at nucleotide 1089 that improved the AIC by 60 units. The trees for each partition were then rooted and branch-re-scaled using TreeTime, and the resulting tanglegram was rendered using dendextend. As can be seen above, the recombination is all between closely related sequences and does not alter the relative position of the 1984, 1992, 2001, 2008, and 2016 spikes used in the experiments. See https://github.com/jbloomlab/CoV_229E_antigenic_drift/blob/master/results/gard_tanglegram.md for details of the analysis steps described above. (TIF) [file ppat.1009453.s002.tif]

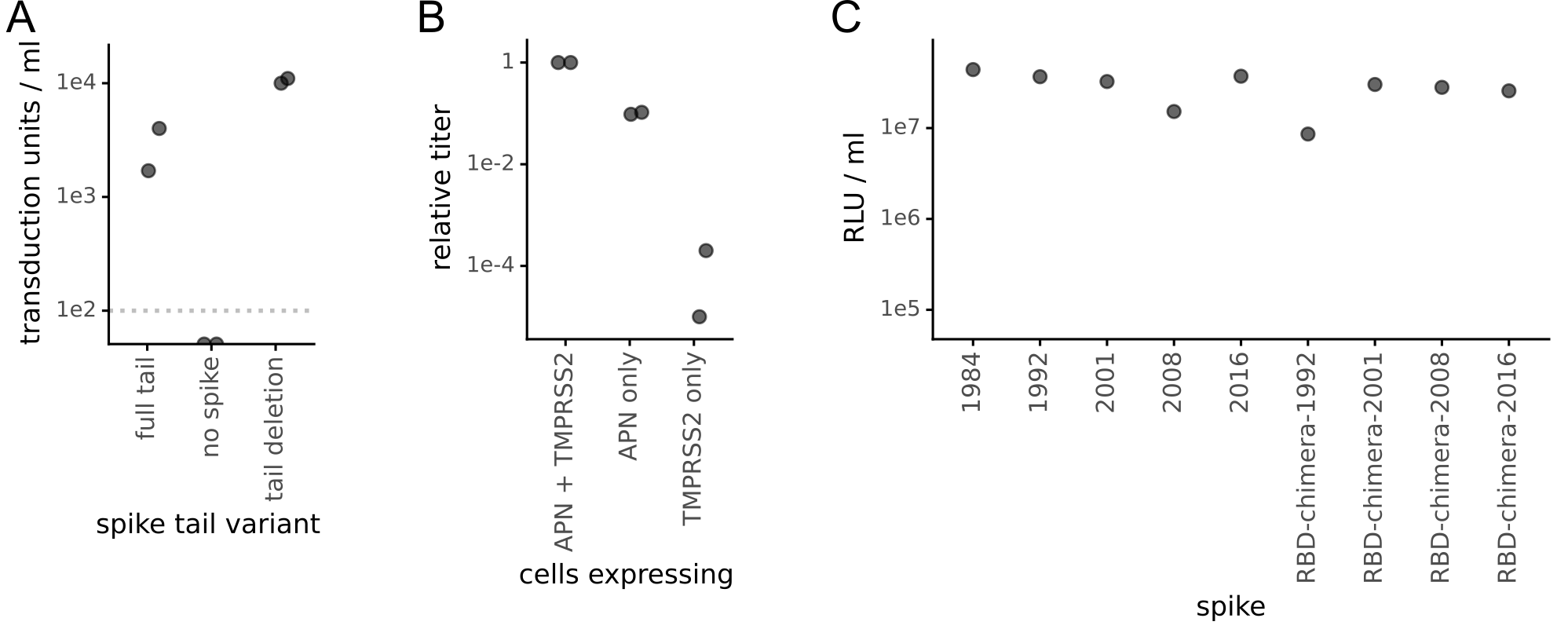

Supplement: S3 Fig — (A) Titer in transduction units per ml as determined using flow cytometry of lentiviral particles pseudotyped with the full-length 2016 spike or that spike with a deletion of the last 19 residues in spike (the end of the cytoplasmic tail) on 293T cells transfected with a plasmid expressing APN. The dotted gray line is the limit of detection, and the titers in the absence of spike were below this line (undetectable). (B) Efficient entry by the pseudotyped virions depends on expression of APN and to a lesser extent TMPRSS2. Virions pseudotyped with the 2016 spike with the C-terminal deletion were infected into 293T cells transfected with plasmids expressing one or both of APN and TMPRSS2, and titers were determined by luciferase luminescence. Titers are normalized to one. (C) All of the 229E spikes and chimeras used in this study mediated efficient viral entry. Lentiviral particles were pseudotyped with the indicated spike (in all cases with the C-terminal deletion) and titers were determined using luciferase luminescence on 293T cells transfected with plasmids expressing APN and TMPRSS2. (TIF) [file ppat.1009453.s003.tif]

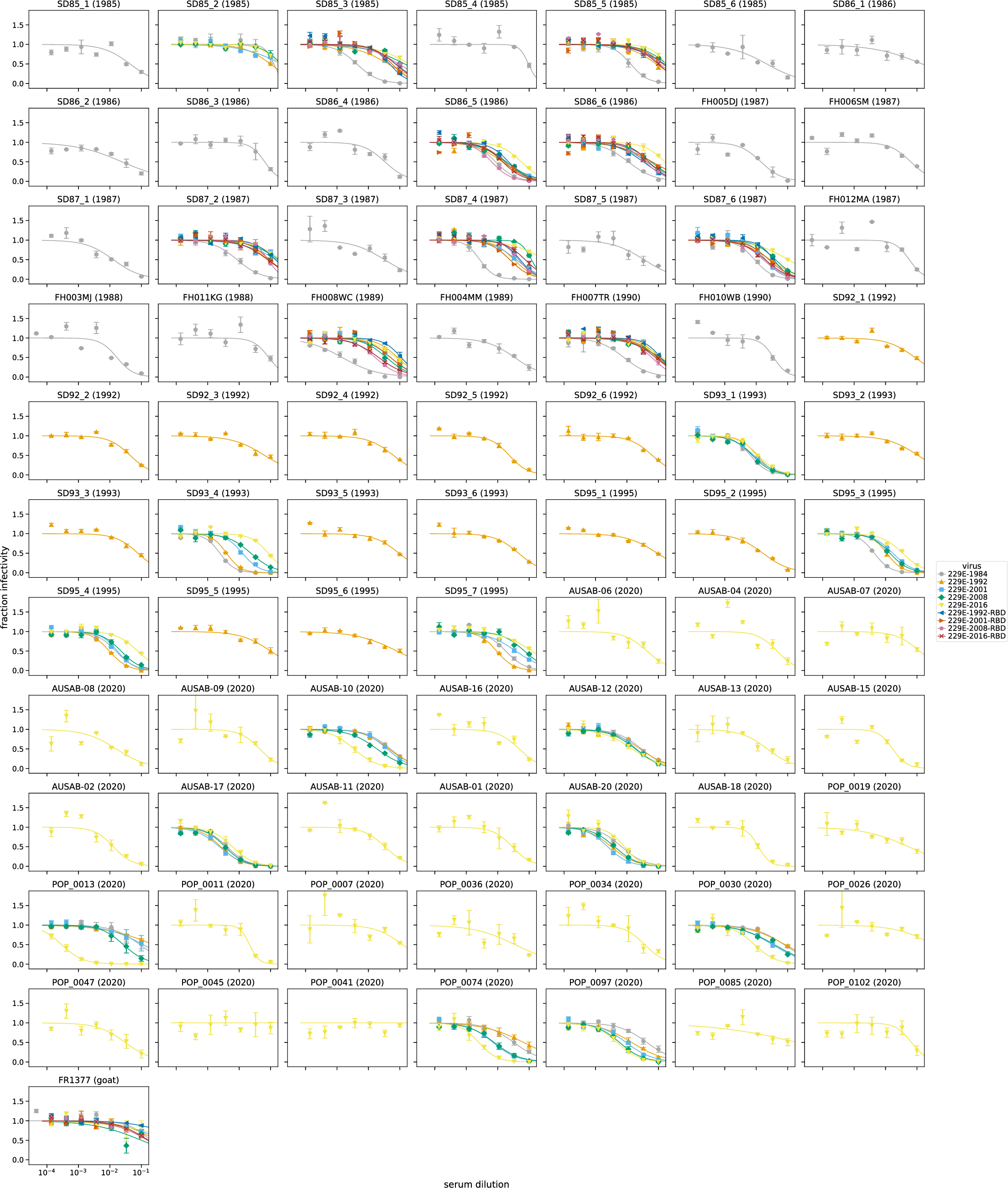

Supplement: S4 Fig — Each facet is a serum, with titles indicating the year the serum was collected. Each point is the fraction infectivity at that serum concentration averaged across at least two replicates (error bars are standard error), with colors indicating the virus. The fits are 2-parameter Hill curves with baselines fixed to 1 and 0, and were fit using neutcurve (https://jbloomlab.github.io/neutcurve/). IC50s are in S3 Data. The curves are also at https://github.com/jbloomlab/CoV_229E_antigenic_drift/blob/master/exptl_data/results/all_neut_by_sera.pdf (TIF) [file ppat.1009453.s004.tif]

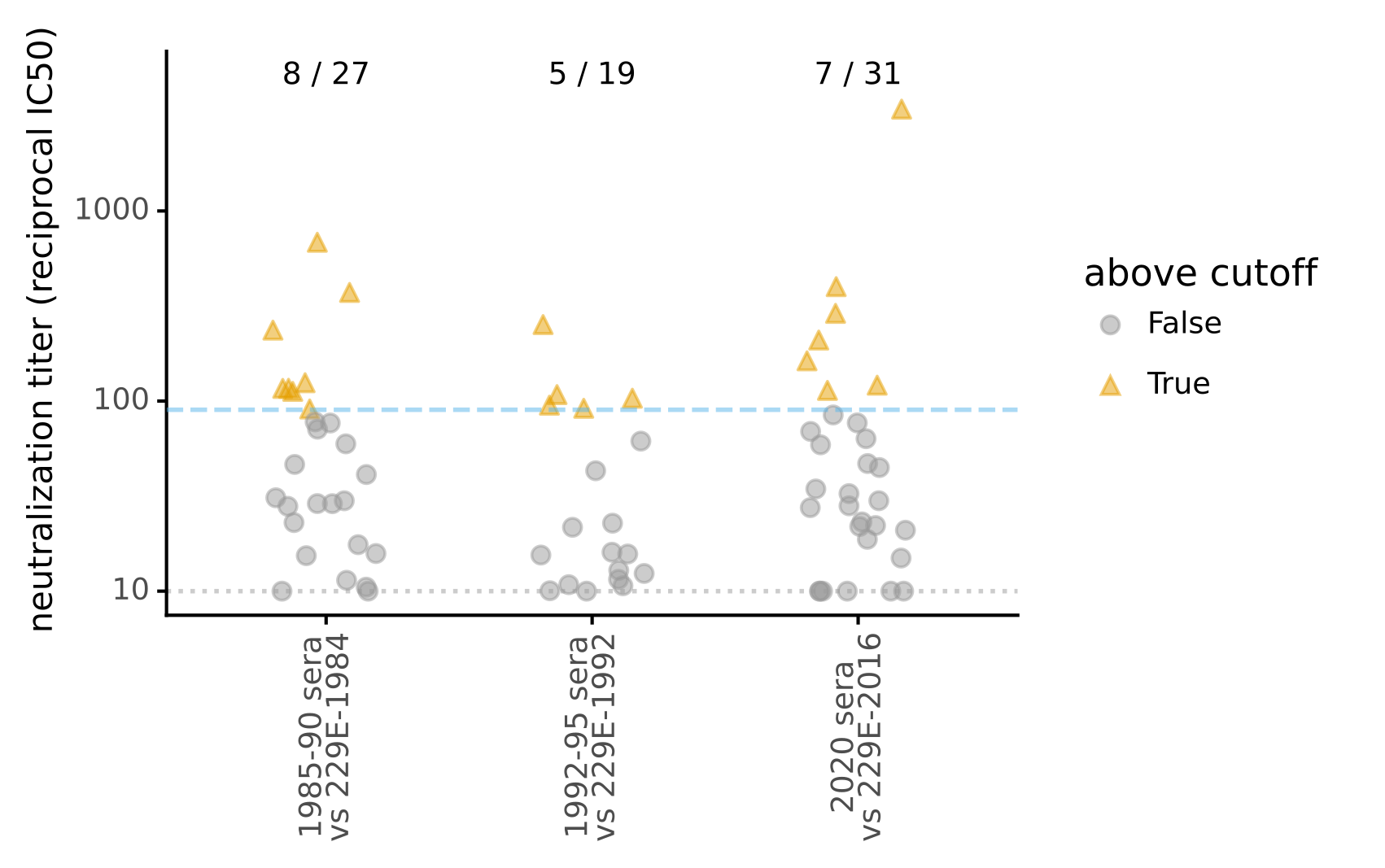

Supplement: S5 Fig — Each sera was tested against the most-recent virus isolated prior to the serum collection date: in other words, sera collected between 1985–1990 was tested against the 1984 spike, sera collected between 1992–1995 was tested against the 1992 spike, and sera collected in 2020 was tested against the 2016 spike. Each point shows the neutralizing titer for a different serum (see S4 Fig for full neutralization curves). Sera above the cutoff of 1:90 (blue dashed line) was then used for further studies against the full panel of viruses (e.g., Figs 2, 3, and 4). The numbers at the top of the plot indicate the number of sera above the cutoff out of the total sera tested in each timeframe. The dotted horizontal line at the bottom of the plot is the lower limit of detection of the neutralization assay. Quantitative neutralization titers for each sera are in S3 Data. (TIF) [file ppat.1009453.s005.tif]

RBD only (143 sites)

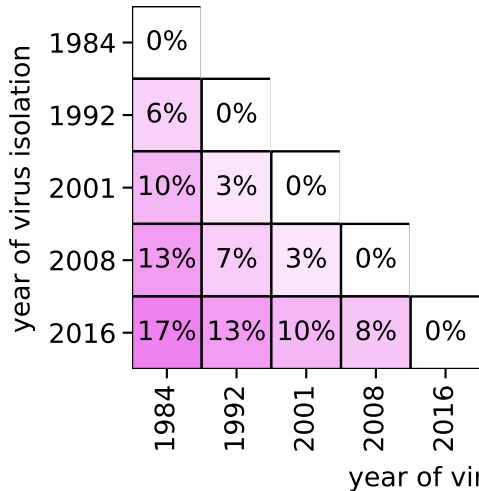

full spike (1154 sites)

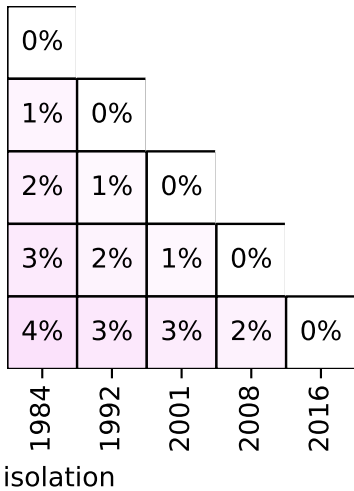

Supplement: S2 Data — There are nine sequences: the five spikes from the 1984, 1992, 2001, 2008, and 2016 viruses (named by strain as shown in Fig 1A), and the four chimeras that consist of the 1984 spike with the RBD of each of the other strains. Each GenPept file annotates key domains in the spike. Note that the C-terminal 19 amino acids are deleted off each spike. These files are at https://github.com/jbloomlab/CoV_229E_antigenic_drift/tree/master/results/seqs_for_expts (ZIP) [file ppat.1009453.s007.zip › S2_Data/n_aa_diffs.pdf]
